# Combined Effect of Temperature and Different Light Regimes on the Photosynthetic Activity and Lipid Accumulation in the Diatom *Phaeodactylum tricornutum*

**DOI:** 10.3390/plants14030329

**Published:** 2025-01-22

**Authors:** Encarnación Díaz-Santos, Luis G. Heredia-Martínez, Luis López-Maury, Manuel Hervás, José M. Ortega, José A. Navarro, Mercedes Roncel

**Affiliations:** 1 Instituto de Bioquímica Vegetal y Fotosíntesis (IBVF), cicCartuja, Universidad de Sevilla and CSIC, 41092 Seville, Spain; ediaz6@us.es (E.D.-S.); lheredia@us.es (L.G.H.-M.); llopez1@us.es (L.L.-M.); mhervas@us.es (M.H.); ortega@us.es (J.M.O.); jnavarro@ibvf.csic.es (J.A.N.); 2 Departamento de Bioquímica Vegetal y Biología Molecular, Facultad de Biología, Universidad de Sevilla, 41012 Seville, Spain

**Keywords:** lipids, PAM fluorescence, *Phaeodactylum tricornutum*, photosynthesis, temperature, thermoluminescence, triacylglycerides

## Abstract

The aim of this study was to investigate the combined effects of temperature and light on the photosynthetic parameters and lipid accumulation in the diatom *Phaeodactylum tricornutum*, a model organism widely used for studies on diatom physiology, ecology, and biotechnology. Our results highlight the importance of the interaction between temperature and light intensity in influencing growth rates, pigments and active photosystems content, photosynthetic efficiency, lipid production and fatty acid composition in *P. tricornutum*. Measurements of the maximum electron transport rate (rETR_max_) and rETR at maximum PAR (830 µmol m^−2^ s^−1^) confirmed that *P. tricornutum* exhibits significantly higher light sensitivity as growth temperature increases under light/dark cycles at two light intensities (25–60 µmol m^−2^ s^−1^). However, this trend was reversed under continuous light (25 µmol m^−2^ s^−1^). Moreover, higher rETR_max_ values (up to double) were observed at higher irradiance, either in intensity or under continuous light regimes, at the two temperatures tested. On the other hand, increasing light intensity amplified the observed effect of temperature on photosystem I (PSI) activity under light/dark regimes, but not under continuous light conditions. This resulted in a greater deficiency in PSI activity, likely due to limitations in electron supply to this photosystem. Furthermore, increasing the culture temperature from 20 °C to 25 °C triggered an increase in the number and size of cytoplasmic lipid droplets under conditions of increased light intensity, with an even more pronounced effect under continuous illumination. Notably, the combination of 25 °C and continuous illumination resulted in a more than twofold increase in triacylglyceride (TAG) content, reaching approximately 17 mg L^−1^. This condition also caused a substantial rise (up to ≈90%) in the proportions of palmitoleic and palmitic acids in the TAG fatty acid profile.

## 1. Introduction

Diatoms are a group of eukaryotic microalgae found in marine and freshwater habitats. They are considered both the dominant life form in oceanic phytoplankton and the largest group of biomass producers on Earth [1]. In addition to their key role in the global carbon cycle and ecological importance, several diatom strains represent a promising option as feedstock for producing biodiesel and high-value-added compounds [2]. Diatoms are also used in aquaculture as a feed source due to their highly valued nutritional profile, which includes a high protein to carbohydrate ratio and elevated levels of highly unsaturated fatty acids (HUFAs) [3,4].

Environmental factors, such as temperature and light intensity, can influence the growth, survival, and distribution of diatoms, as well as their biotechnological performance [5,6]. Diatoms have an optimal temperature range for growth, and temperatures outside this range can lead to reduced growth rates or even death [7]. Studies on temperature tolerance in diatoms have been conducted to understand their response to thermal stress in terms of cell growth and productivity, including the effects of temperature on photosynthesis, growth rates, cell size and nutrient uptake [7,8,9,10,11].

*Phaeodactylum tricornutum* is a coastal marine diatom used as a model organism for studies on diatom physiology and ecology, as well as in biotechnology and biofuel production [12,13]. The optimal temperature for the growth of *P. tricornutum* is approximately 20 °C, with growth rates declining significantly above and below this value [7,14]. Several studies have investigated the temperature tolerance of *P. tricornutum*, focusing on the effects of temperature on growth rates, lipid accumulation, and photosynthetic efficiency [7,10,11].

Temperature changes influence membrane lipid production and composition in diatoms. In general, there is an inverse relationship between temperature and the degree of lipid desaturation [10,15]. At low temperatures, *P. tricornutum* increases the unsaturation of its membrane lipids to maintain membrane fluidity, while at elevated temperatures, an increase in the saturation of the fatty acids in cellular and thylakoid membranes is observed [9,10,15,16]. In addition to lipid metabolism, temperature also affects the photosynthetic performance of diatoms. *P. tricornutum* typically achieves efficient photosynthesis within a temperature range close to its optimal growth temperature. However, when this optimal temperature is exceeded, the photosynthetic rate declines [10]. This limitation results in excess light energy, leading to photoinhibition, which primarily damages photosystem II (PSII), the most thermosensitive component of the photosynthetic apparatus [17]. The reported transcriptomic response of *P. tricornutum* to elevated temperatures has shown that this organism downregulates genes involved in photosynthesis, while activating signaling pathways related to fatty acid and nitrogen metabolism [11].

Many of the response mechanisms to temperature changes in microalgae are also observed in their reaction to high light levels [5]. It is well established that the growth rate increases with increasing light until the maximum growth rate is reached, after which growth may decline due to photoinhibition [18]. In this context, *P. tricornutum* has developed several strategies to cope with excess light stress. These adaptations include the following: (i) adjustment of the photosynthetic apparatus by altering the composition and activity of the photosynthetic complexes to protect the photosynthetic machinery from light-induced damage; (ii) activation of non-photochemical quenching (NPQ) mechanisms to dissipate excess light energy as heat, preventing the formation of reactive oxygen species (ROS); (iii) enhanced PSII repair; and (iv) predominance of cyclic electron flow to optimize ATP production while minimizing the risk of ROS production [2,19]. Additionally, high light conditions are known to trigger the accumulation of neutral lipids, primarily in the form of triacylglycerides (TAGs), which are stored as reserve lipids in lipid droplets (LDs) [20,21,22]. In this way, diatoms redirect carbon metabolism towards the production of energy-rich lipids, which accumulate within LDs and can be rapidly degraded and recycled when conditions improve [21,22].

The effects of temperature and light on marine diatoms have been studied and characterized independently; however, fewer studies have examined the combined effects of temperature and light interactions on these organisms [23,24]. It has been shown that the photosynthetic response to temperature depends on available light (both in intensity and type), with different responses being observed at sub-saturating light levels compared to saturating levels [25]. In fact, some studies have demonstrated that the photosynthetic apparatus exhibits greater thermal stability at low irradiances than at high irradiances [26].

In this study, we investigated the combined effects of temperature and light under both light–dark cycles and continuous light conditions on photosynthetic activity in the model marine diatom *P. tricornutum*. Illumination intensities intended to approximate natural growing conditions were used. First, an illumination level of 25 µmol m^−2^ s^−1^ was chosen to represent moderate radiation conditions [10]. Second, a light intensity of 60 µmol m^−2^ s^−1^ was also used, which is close to light-saturating conditions ([10,27] and see Table 1). Additionally, the accumulation of neutral lipids—primarily in the form of TAGs stored in LDs—was also investigated, as *P. tricornutum* is considered a potential resource for biodiesel production.

## 2. Results

### 2.1. Influence of Temperature Under Two Different Light Intensities in Light/Dark Regime

The growth of *P. tricornutum* cells was first characterized under our experimental conditions. Cells were cultured under light/dark cycles at two light intensities—25 µmol m^−2^ s^−1^ (standard light conditions, SL) and 60 µmol m^−2^ s^−1^ (higher light conditions, HL), and at two different temperatures—20 °C and 25 °C, corresponding to optimal and high temperature conditions, respectively [14]. A decrease in growth was observed at 25 °C compared to 20 °C under both SL and HL conditions, as evidenced by lower values for final cell concentration (cells per mL of culture) and specific growth rates (µ) (Table 1). This decrease in growth was more pronounced under SL conditions (≈25% reduction in both cell concentration and µ) compared to HL conditions (≈10–20% reduction in both cells concentration and µ [Table 1]). Notably, HL conditions at both 20 °C and 25 °C promoted only a slight increase in *P. tricornutum* cell growth compared with SL conditions (Table 1). This indicates that this light intensity (60 µmol m^−2^ s^−1^) is already near light saturation under our culture conditions, as previously reported [27]. For the culture conditions investigated here, biomass—measured as cell number—ranged from ≈1 to 2 × 10^7^ cells mL^−1^, with a dry weight of ≈300–400 μg mL^−1^ (Table 1). Although the measurement error in the dry weight data prevents definitive conclusions, the data suggest a relative increase in the dry weight of the cultures at 25 °C, despite the lower number of cells (Table 1, and see below).

It has been documented that *P. tricornutum* cells exposed to warmer temperatures exhibit reduced photosynthetic efficiency but increased levels of photosynthetic pigments [10]. Consistent with this, the per-cell content of total Chl and carotenoids increased when the temperature was raised from 20 °C to 25 °C under both SL and HL conditions (Table 1). While this increase was minimal under SL conditions, a temperature shift from 20 °C to 25 °C under HL conditions led to a substantial rise in total Chl (up to 135%) and carotenoids levels (up to 200%) per cell (Table 1).

Diatoms contain Chl *a* as the primary pigment for photosynthesis, while Chl *c* serves as an accessory pigment within the light-harvesting antennae [28]. The Chl *a*/Chl *c* ratio thus reflects the balance between the photosynthetic reaction centers and the antenna complexes, varying with light availability in response to low-light conditions or higher light intensities [28]. As shown in Table 1, the absolute values for this ratio were approximately 140% higher under HL conditions than under SL. Additionally, under both SL and HL conditions, the temperature increase from 20 °C to 25 °C led to a similar rise in the Chl *a*/Chl *c* ratio (approximately 1.4 to 1.5 times).

The global photosynthetic activity of the cultures under SL and HL conditions was assessed by measuring net oxygen evolution (Table 1). As expected, the highest net photosynthetic activity per cell was recorded at the optimal temperature of 20 °C compared to 25 °C. Under SL conditions, a more significant decrease (up to ≈60%) in normalized net photosynthetic activity per cell was observed at 25 °C compared to 20 °C. In contrast, under HL conditions, the decline in photosynthetic activity was less pronounced, with activity levels remaining at up to ≈80% of those observed at 20 °C (Table 1).

**Figure 1:**
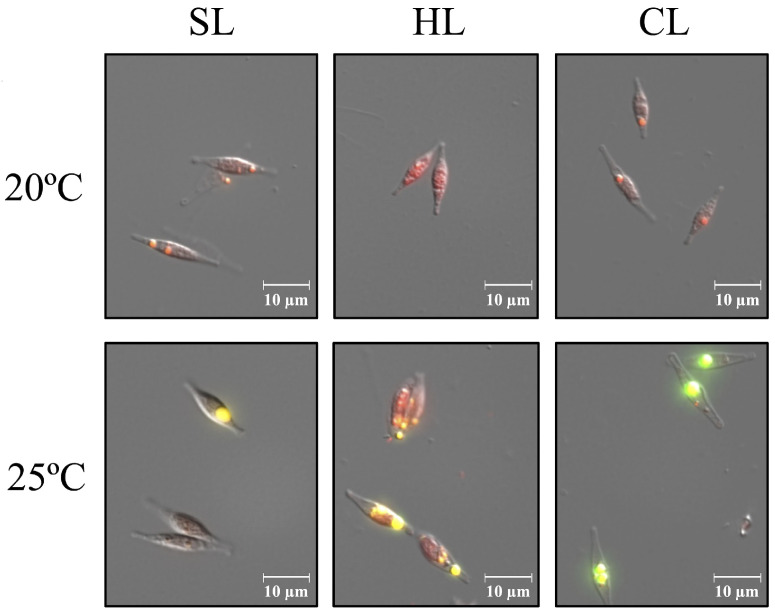
Fluorescent microscopy images of *P. tricornutum* cells stained with Nile Red, from cultures grown under different temperature and light intensity conditions, as indicated. SL, light/dark cycle illumination at 25 µmol m^−2^ s^−1^; HL, light/dark cycle illumination at 60 µmol m^−2^ s^−1^; CL, continuous illumination at 25 µmol m^−2^ s^−1^. For further details, see Section 4.

Changes in cell morphology at the two different temperatures and light intensities were studied in vivo using fluorescence microscopy (Figure 1). Morphological analyses of cells cultured under SL conditions, compared to those under HL conditions, revealed no significant differences in cell morphology or size at the different temperatures and light intensities (Figure 1). In both cases, the typical fusiform morphotype was predominantly observed, although cells grown at 25 °C occasionally showed the formation of small internal granules, which were more abundant under HL conditions (Figure 1). These granules are likely lipid droplets (LDs) (Figure 1, and see below), as it has been previously reported that *P. tricornutum* increases lipid accumulation under stress conditions [21,22].

To further assess lipid production, the accumulation of neutral lipids stored as LDs was qualitatively analyzed in cells cultured under SL and HL conditions at the two tested temperatures. Using Nile Red (NR) staining and fluorescence microscopy (Figure 1) [29], neutral lipids content was visualized, with fluorescence intensity correlating with lipid content. Higher yellow fluorescence indicates a greater accumulation of neutral lipids [30]. Although cells grown at 20 °C showed some fluorescence associated with NR staining and the presence of LDs, fluorescence intensity was significantly higher at 25 °C under both SL and HL conditions, indicating a marked increase in neutral lipids accumulation (Figure 1). The increase in lipid content in LDs may help explain the relative increase in dry weight observed in cultures at this temperature, despite the lower cell density, as noted previously (Table 1).

The combined effects of temperature and light intensity on the photosynthetic activity of *P. tricornutum* cells were further investigated. First, the effects of temperature on PSII activity were assessed by measuring Chl *a* fluorescence using a DUAL-PAM fluorometer. As shown in Table 1, a moderate but statistically significant (*p*-values < 0.05) increase in the maximum quantum yield of PSII, F_v_/F_m_, was observed with increasing temperature under both SL and HL conditions.

**Figure 2:**
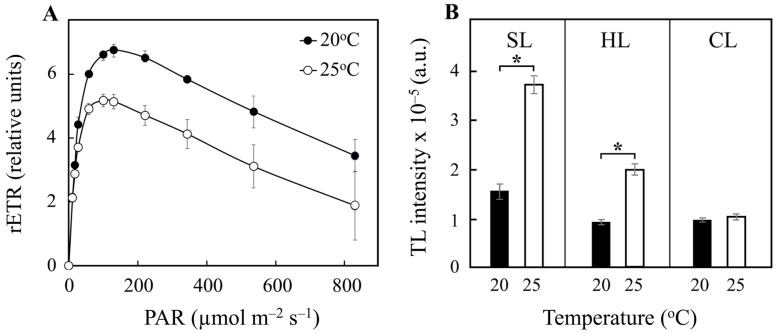
(**A**) Relative linear electron transport rate (rETR) in *P. tricornutum* cultures under light/dark cycle illumination with standard light (SL; 25 µmol m^−2^ s^−1^) and at 20 °C or 25 °C, as indicated. rETR values were determined as a function of irradiance derived from steady-state light curves. Chlorophyll fluorescence was measured using a pulse–amplitude modulation fluorometer, and rETR values were determined during stepwise increases in photosynthetically active radiation (PAR) from 0 up to 830 µmol m^−2^ s^−1^ light intensity. Data represent mean values ± SD of five independent measurements. (**B**) Intensities of the TL B-band for *P. tricornutum* cultures investigated at 20 °C (black bars) or 25 °C (white bars) under different light intensity conditions, as indicated (SL, light/dark cycle illumination at 25 µmol m^−2^ s^−1^; HL, light/dark cycle illumination at 60 µmol m^−2^ s^−1^; CL, continuous illumination at 25 µmol m^−2^ s^−1^). Intensities were obtained from the component analysis of the TL glow curves. Data represent mean values ± SD of five independent measurements. Asterisks mark statistically significant differences between data groups (*p* < 0.05). For additional details, see Section 4.

Rapid light curves (RLCs) of cell cultures under SL and HL illumination at 20 °C and 25 °C were derived, with Figure 2A showing the data obtained under SL conditions as an example. In the RLCs, as cells were exposed to gradually increasing light intensities, the relative electron transport rate (rETR), which represents the ratio between absorbed light quanta and transported electrons, increased in parallel up to its maximum capacity, corresponding to the maximum electron transport rate (rETR_max_; Figure 2A and Table 1). Beyond this point, increasing light intensity induced photoinhibition, resulting in a decrease in rETR (Figure 2A) [31,32,33]. As shown in Table 1, the rETR_max_ values calculated from the RLCs analysis were temperature dependent, with values decreasing as the temperature increased from 20 °C to 25 °C under both SL and HL conditions (Table 1). However, the calculated rETR_max_ values were lower under SL conditions compared to HL (≈7 to 5 versus ≈12 to 8; Table 1). A similar pattern was observed when comparing rETR values at the highest light intensity tested (PAR_max_ of 830 µmol m^−2^ s^−1^), with a decrease in these values as the temperature increased from 20 °C to 25 °C under both SL and HL conditions (Table 1). Again, rETR values (at PAR_max_) were lower under SL conditions compared to HL (from ≈3.4 to 1.9 versus ≈11.3 to 6.8; Table 1). Taken together, these results confirm a substantially higher rETR_max_ at higher irradiance intensities at each temperature for *P. tricornutum*, but also a significantly higher light sensitivity as growth temperature increased.

The effect of temperature on the electron transfer activity of PSII in *P. tricornutum* cells cultured under SL and HL conditions was also studied using the thermoluminescence (TL) technique (Figure 2B and Supplementary Figure S1). Under SL conditions, the excitation of *P. tricornutum* cells with two flashes at 1 °C induced the appearance of TL glow curves, with significant differences in signal intensity depending on the culture temperature (Supplementary Figure S1). A similar significant decrease in the total TL signal intensity (of ≈50–60%) was observed in cells grown at 20 °C compared to those grown at 25 °C under both SL and HL conditions (Figure 2B). However, the signal intensities at each temperature were significantly higher (≈2 times) under SL conditions compared to HL (Figure 2B).

**Figure 3:**
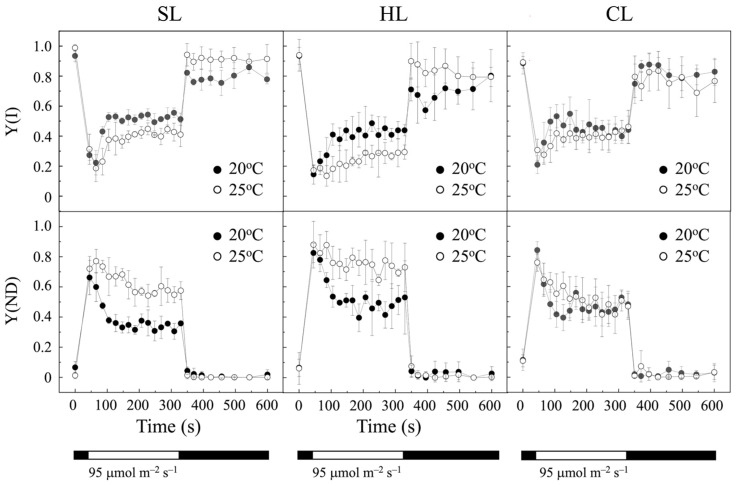
PSI activity of *P. tricornutum* cells grown under different temperature and light intensity conditions, as indicated. The redox state of the PSI reaction center, P700, was monitored through changes in absorbance at 830 nm versus 875 nm, measured with a pulse–amplitude modulation fluorometer. Cultures were kept in the dark for 30 min prior to the measurements. After the initial determination of the maximal oxidation of P700, actinic light was turned on at an intensity of 95 µmol m^−2^ s^−1^ and saturating pulses were applied every 20 s. After 5 min, the actinic light was switched off, and measurements continued for another 5 min. Changes in (upper) quantum yields of PSI, Y(I), and (lower) donor-side limitations, Y(ND), during the induction curve are displayed. Data represent the mean values ± SD of five independent measurements. The white and black bars below graphs indicate periods of illumination with actinic light and darkness, respectively. SL, light/dark cycle illumination at 25 µmol m^−2^ s^−1^; HL, light/dark cycle illumination at 60 µmol m^−2^ s^−1^; CL, continuous illumination at 25 µmol m^−2^ s^−1^.

The effect of temperature on PSI activity in *P. tricornutum* cells cultured under SL and HL conditions was also investigated by measuring changes in the P700 redox state during illumination (Figure 3), as previously described [32,33]. In dark-adapted cultures, P700 is reduced because the acceptor side of P700—i.e., the Calvin–Benson cycle and subsequent reactions—is inactive. Under actinic light, P700 is initially oxidized and then re-reduced by electrons from the plastoquinone (PQ) pool. By applying saturating light pulses, the ability of P700 to be oxidized and re-reduced can be assessed [32,33]. Induction–recovery curves were first performed on cell cultures grown at both 20 °C and 25 °C under SL conditions, revealing a decrease in the quantum yield of PSI photochemistry, Y(I), at 25 °C compared to 20 °C (Figure 3, upper). Additionally, a higher degree of donor-side limitations, Y(ND), was also observed in cells cultured at 25 °C compared to 20 °C (Figure 3, lower). The lower Y(I) in cells cultured at 25 °C suggests a loss of PSI activity due to the limited availability of electron donors to PSI under lower light conditions [32,33]. In contrast, under HL conditions, a lower Y(I) and a higher Y(ND) were observed compared to the SL condition at both 20 °C and 25 °C (Figure 3), with a more pronounced effect at 25 °C. The lower Y(I) observed in cells cultured under HL conditions indicates a deficiency in PSI activity, likely due to limitations in providing electrons to this photosystem, probably induced by the higher irradiance. Thus, increasing light intensity exacerbates the temperature-induced effects on PSI activity by further limiting the availability of electrons to PSI under higher light. In contrast, small and similar acceptor-side limitations, Y(NA), were observed under all temperature and illumination conditions tested.

The maximal P700^+^ signal upon full oxidation (P_m_) was measured under both SL and HL conditions, as previously described [32,33]. After illumination with FR light followed by a saturating light pulse, P700 became oxidized and reached a maximal level of P700^+^. The amplitude values obtained show that P_m_ increased under both light conditions when the temperature was raised from 20 °C to 25 °C (Table 1). Thus, the observed deficiency in PSI activity does not correlate with a lower amount of photochemically active PSI centers. Additionally, P_m_ exhibited higher absolute values under SL conditions compared to HL, particularly at 25 °C, with values approximately 1.7 times higher (Table 1). These results suggest that, at higher irradiance and lower temperature, there is a lower amount of photochemically active PSI in *P. tricornutum*.

### 2.2. Influence of Temperature Under Continuous Light

The responses of cells cultured at 20 °C and 25 °C under CL conditions were analyzed. A comparison between SL and CL conditions (both at 25 µmol m^−2^ s^−1^) revealed specific differences in the response to the temperature increase from 20 °C to 25 °C under constant illumination. Although both light regimes showed a similar decrease in µ with increasing temperature (Table 1), some distinct effects were observed under CL conditions. For instance, Chl *c* content was similarly high under CL at both 20 °C and 25 °C compared to SL conditions (≈1.7 times higher; not shown). This resulted in a decrease in the Chl *a*/Chl *c* ratio under CL conditions, particularly at 25 °C (Table 1), suggesting a reduced ratio of photosynthetic reaction centers to antenna complexes. Additionally, carotenoid content decreased drastically under CL conditions as the temperature increased, whereas carotenoid levels remained stable under SL conditions (Table 1).

The morphological analysis of cells cultured under CL conditions, compared to SL conditions, showed no significant differences in cell morphology or size at the different temperatures and light intensities (Figure 1). Furthermore, no differences in LD size were observed in representative fluorescence microscopy images (using NR staining) of cells cultured at 20 °C under SL and CL conditions (Figure 1). However, cells cultured at 25 °C under CL conditions exhibited a pronounced increase in NR fluorescence and a large number of large LDs, indicating a significant accumulation of neutral lipids (Figure 1, and see below).

The responses of PSII activity of *P. tricornutum* cells grown at 20 °C and 25 °C under CL conditions were also assessed by measuring Chl *a* fluorescence. As shown in Table 1, the F_v_/F_m_ values under continuous light were slightly lower than those observed under SL conditions, but they remained unchanged with increasing temperature, in contrast to the increase observed under SL conditions. This is particularly evident at 25 °C under CL conditions, where F_v_/F_m_ is lower compared to SL conditions (0.58 versus 0.66; Table 1). However, rETR_max_ was higher under CL than under SL conditions at both 20 °C and 25 °C, with a value approximately 2 times higher at 25 °C (≈10.5 versus 5.2; Table 1). An interesting observation is that rETR_max_ values decrease with increasing temperature from 20 °C to 25 °C under SL and HL conditions, while they increase with temperature under CL conditions (Table 1). A similar pattern was observed when rETR values at PAR_max_ were compared; values decreased with increasing temperature from 20 °C to 25 °C under SL and HL conditions, but they increased under CL conditions (Table 1). As a result, the absolute values for rETR at PAR_max_ were significantly higher (2 to 7 times) under CL compared to SL conditions (Table 1). On the other hand, the signal intensity of the TL glow curves obtained in *P. tricornutum* cells grown under CL was similarly low for both temperatures, in contrast to the increase observed at higher temperature under SL and HL conditions (Figure 2B).

Induction–recovery curves were also developed to analyze the response of PSI activity in *P. tricornutum* cells cultured at 20 °C and 25 °C under CL conditions (Figure 3). Interestingly, in contrast to what was observed under light/dark cycles, both in SL and HL conditions, no significant changes were observed in Y(I) or Y(ND) when the temperature was increased from 20 °C to 25 °C under CL conditions (Figure 3). However, as in SL and HL conditions, a low Y(NA) was observed (data not shown). Overall, the imbalance caused by continuous light exposure appears to counteract the effect of the temperature increase.

The P_m_ values of *P. tricornutum* cells cultured at 20 °C and 25 °C under CL conditions were also measured. Table 1 shows that these P_m_ values increased when the temperature was raised from 20 °C to 25 °C, consistent with the changes observed previously under SL (and HL) conditions (Table 1). However, P_m_ values were lower under CL compared to SL conditions, particularly at 25 °C (Table 1). In fact, the P_m_ values obtained under CL and HL conditions were similar at each temperature (Table 1). These results suggest that higher irradiance (either in terms of intensity or exposure duration) and temperature lead to a lower amount of photochemically active PSI in *P. tricornutum*.

### 2.3. Combined Effect of Temperature and Light Regimes on Lipid Content and Fatty Acid Composition

The appearance of lipid droplets, as observed using the NR staining, indicates lipid accumulation in *P. tricornutum* cells under the conditions of increased temperature and illumination, particularly under HL and CL conditions at 25 °C (Figure 1). *P. tricornutum* has significant commercial potential due to its high lipid content [12,13]. Consequently, we performed a more detailed analysis of the changes in lipid content and composition in *P. tricornutum* cells grown under SL, HL and CL illumination at 20 °C and 25 °C.

**Figure 4:**
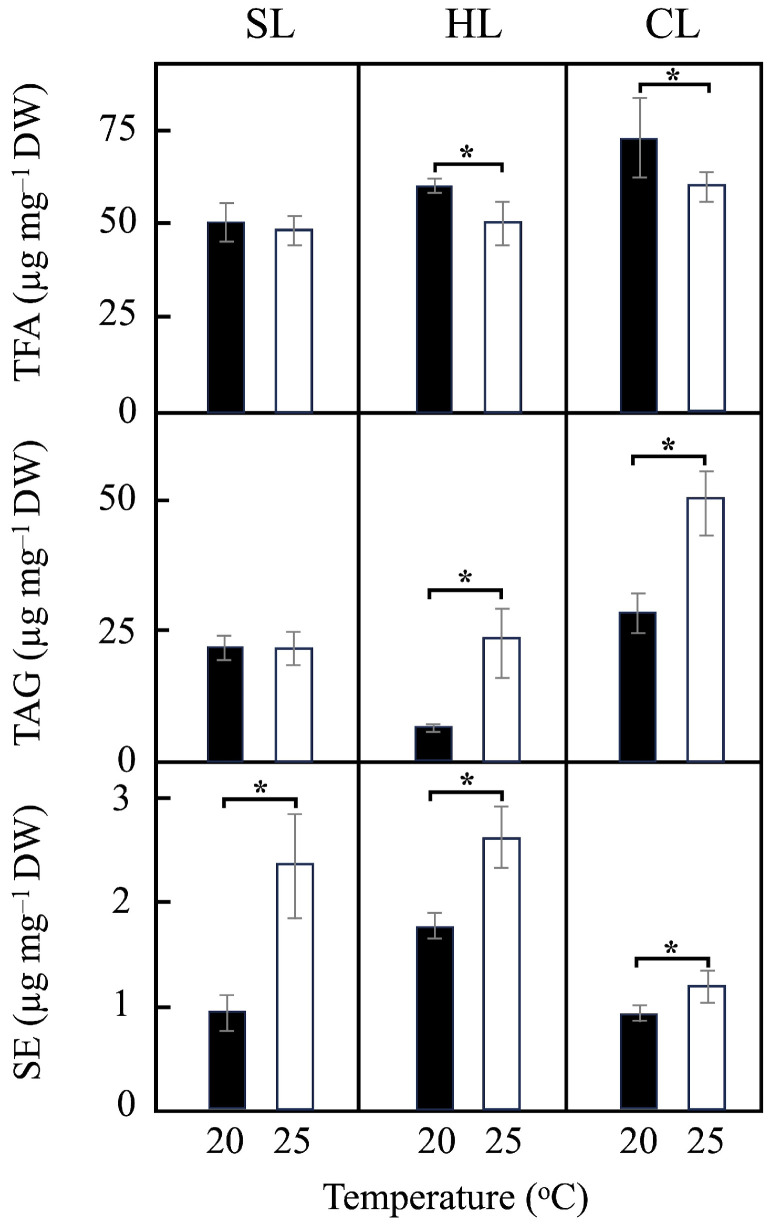
Total fatty acids (TFA), triacylglycerides (TAG) and sterol esters (SE) contents in *P. tricornutum* cultures. Final TFA, TAG and SE contents (measured as µg per mg of dry weight) were determined after 15 days of cultivation of *P. tricornutum* cells grown under 20 °C (black bars) or 25 °C (white bars) and different light regimes, as indicated. Data represent the mean values ± SD of three independent biological replicates. SL, light/dark cycle illumination at 25 µmol m^−2^ s^−1^; HL, light/dark cycle illumination at 60 µmol m^−2^ s^−1^; CL, continuous illumination at 25 µmol m^−2^ s^−1^. Asterisks mark statistically significant different data groups (*p* < 0.05) (see Section 4).

Figure 4 presents the total fatty acids (TFA), triacylglycerides (TAG), and sterol esters (SE) contents relative to the culture dry weight (DW) in *P. tricornutum* cells grown at 20 °C and 25 °C under the different light regimes. Additionally, total TFA, TAG, and SE contents, expressed in mg per liter, were also determined (Supplementary Figure S2), showing similar profiles to those depicted in Figure 4. Under SL conditions, the TFA content exhibited no substantial variations with temperature. In contrast, under HL and CL conditions, a decrease in the TFA content was observed in cells grown at 25 °C compared to those grown at 20 °C, as shown in Figure 4. Additionally, at each temperature, the TFA content increased with increasing irradiance (either in terms of intensity or illumination time) (Figure 4). The most notable effect of the light regime change was an approximate 40% increase in TFA content in cells grown at 20 °C under CL conditions compared to SL conditions (Figure 4). Regarding TAG content, an increase was observed in cells grown at 25 °C compared to 20 °C under both HL and CL conditions (Figure 4 and Supplementary Figure S2), consistent with NR staining observations (Figure 1). In this case, the most significant effect of the change in the light regime was a more than twofold increase in TGA content at 25 °C under CL conditions compared to SL conditions. Notably, significant increases in SE content were also detected in cells grown at 20 °C compared to 25 °C under all illumination conditions (Figure 4 and Supplementary Figure S2). It should be noted that additional experiments were conducted at 15 °C; however, these data have been excluded, as the most significant changes in TFA and TAG contents were observed when the culture temperature increased from 20 °C to 25 °C.

The compositions of TFAs in *P. tricornutum* cells cultured under different illumination conditions at 20 °C and 25 °C were quantified as lipid-derived fatty acid methyl esters (FAMEs) using GC-MS (see Section 4) (Supplementary Table S1). Under all tested conditions, the three most abundant fatty acids (with an average content >10%) identified were eicosapentaenoico acid (EPA, C20:5n-3), palmitoleic acid (C16:1c), and palmitic acid (C16:0) (Supplementary Table S1) [2,34]. Our results indicate that increasing the temperature from 20 °C to 25 °C led to an increase in palmitoleic acid (C16:1c) content and a decrease in EPA (C20:5n-3) under light/dark cycle illumination. However, the highest values of palmitoleic acid (C16:1c) were observed under CL conditions (≈26–28 μg mg^−1^ DW). In contrast, the lowest absolute value of EPA content was observed under CL conditions at 25 °C (8.5 μg mg^−1^ DW) (Supplementary Table S1). The presence of docosahexaenoic acid (DHA, C22:6n-3) was confirmed under all tested conditions, with its content basically unaffected by changes in temperature or light regime. Other fatty acids with 18 to 24 carbon atoms were only present in trace amounts (Supplementary Table S1).

**Figure 5:**
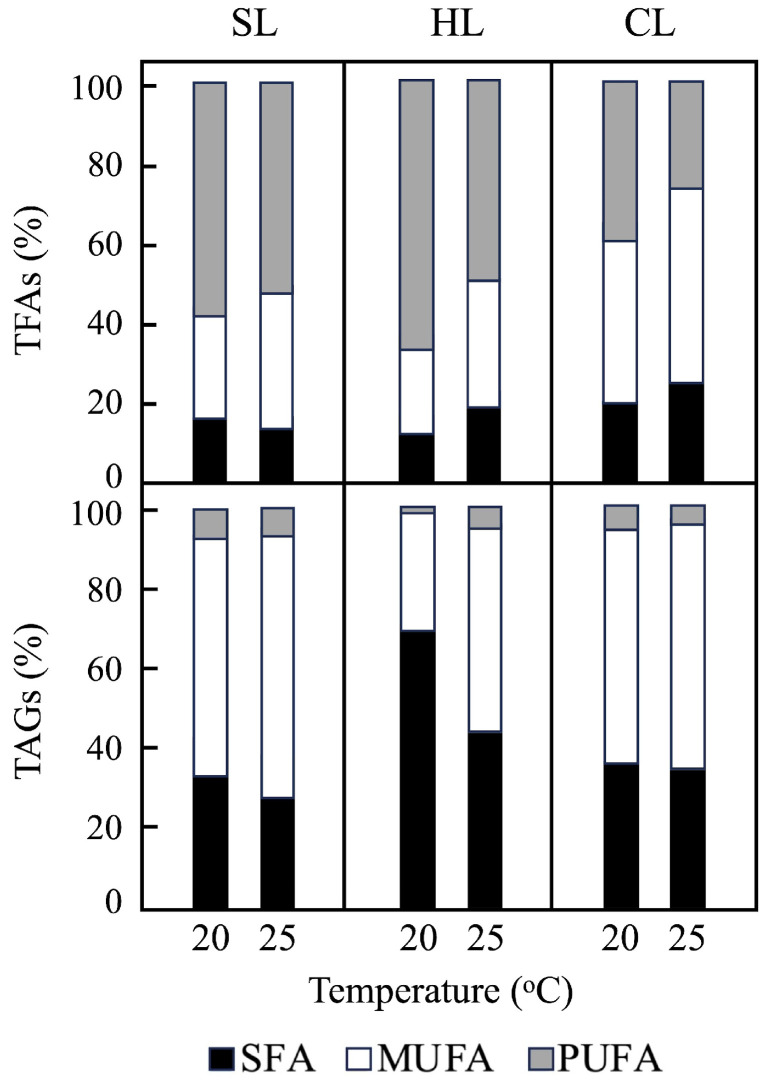
Ratio of saturated (SFA), monounsaturated (MUFA) and polyunsaturated (PUFA) fatty acids contents in *P. tricornutum* cells grown under different conditions of temperature and light intensity, as indicated. Values are expressed as the percentage of total fatty acids (upper; TFAs) or triacylglycerides (lower; TAGs). Data represent mean percentage values from three independent biological replicates. SL, light/dark cycle illumination at 25 µmol m^−2^ s^−1^; HL, light/dark cycle illumination at 60 µmol m^−2^ s^−1^; CL, continuous illumination at 25 µmol m^−2^ s^−1^.

Based on these data, the percentages of saturated, monounsaturated, and polyunsaturated fatty acids (SFAs, MUFAs and PUFAs, respectively) in the total fatty acid content of *P. tricornutum* were calculated (Figure 5, upper). The percentage of PUFAs decreased in parallel with an increase in MUFAs as the temperature rose from 20 °C to 25 °C under all light regimes, while the percentage of SFAs showed only minor changes (Figure 5, upper). However, the lowest values of PUFAs (and the highest values of MUFAs) were observed under CL conditions. Additionally, the ratio of UFAs to SFAs in the TFA content of *P. tricornutum* cells decreased when comparing SL and CL conditions (from ≈6–7 to 3–4; Supplementary Table S1).

Similarly, the fatty acid composition of TAGs in *P. tricornutum* cells was determined (Supplementary Table S2). The data reveal that under the different temperatures and light regimes, the main fatty acids present in TAGs were palmitoleic acid (C16:1c) and palmitic acid (C16:0). However, EPA (C20:5n-3) was found in very small amounts in TAGs (Supplementary Tables S1 and S2), contributing to a drastic decrease in PUFA content (Figure 5, lower). The percentages of SFAs, MUFAs, and PUFAs in total TAGs did not show significant differences under SL and CL conditions at 20 °C and 25 °C, with MUFAs being the predominant component (Figure 5, lower). However, under HL and 20 °C conditions, higher values of SFAs and lower values of MUFAs were detected, with SFAs decreasing and MUFAs increasing at 25 °C (Figure 5, lower). Finally, while the UFAs/SFAs ratio in TAGs increased as the culture temperature rose from 20 °C to 25 °C under SL and HL conditions, this ratio was not affected by temperature under CL conditions (Supplementary Table S2).

## 3. Discussion

### 3.1. Combined Effects of Temperature and Light Regimes on Cell Growth and Photosynthetic Parameters

Light and temperature are critical factors that influence the growth and biochemical composition of microalgae, including diatoms [6,8,35]. While the independent effects of temperature and light on marine diatoms have been extensively studied and well characterized [5,6], only a few studies have investigated their combined effects on these organisms [23,24,36]. Temperature can have varying impacts on diatom growth and photosynthetic activity depending on the light regime. In this study, we investigated the combined effects of temperature and light regimes on *P. tricornutum* grown at 20 °C and 25 °C, either under light/dark cycles with two light intensities or continuous light.

Growing *P. tricornutum* at 25 °C, compared to 20 °C, resulted in a decrease in both the specific growth rate and total photosynthetic activity, measured as net O_2_ production, under both light/dark cycles and continuous light (Table 1). A similar reduction in growth rate was observed in previous studies when the temperature was increased from 18 °C to 26 °C [10]. Regarding biomass production in dry weight, our results show average values of approximately 0.4 mg L^−1^ and a cell concentration of about 1.5 × 10^7^ cells mL^−1^. These values are comparable to those observed in other studies under similar culture conditions [37]. However, while no clear conclusions can be drawn regarding the effects of temperature on dry weight biomass production, a slight increase in biomass at 25 °C can be inferred. Our results show a positive correlation between the contents of both total Chl and carotenoids with temperature under light/dark cycles, particularly under HL conditions (Table 1). Higher Chl *a* content has previously been reported in *P. tricornutum* grown at 25 °C, compared to cells grown at 10 °C [38], and at 15 °C compared to 6 °C [39]. The observed increase in the Chl *a*/Chl *c* ratio with temperature under light/dark cycles suggests a shift favoring photochemical energy conversion over accessory pigments, likely as an adaptive response to temperatures exceeding the optimal range. This positive effect of temperature on Chl content contrasts with the negative effect of higher light intensity (Table 1). Specifically, light intensity negatively affected photosynthetic pigment production in *P. tricornutum*, with higher pigment content observed under the lower SL irradiance, which was less favorable for cell growth (Table 1). The higher Chl levels under SL conditions were associated with lower Chl *a*/Chl *c* ratios compared to HL conditions, suggesting that HL favored photochemical energy conversion by increasing the ratio of photosynthetic reaction centers to antenna complexes. In a broader context, a moderate increase in light intensity mitigated the adverse effects of elevated temperature on cell growth and photosynthetic rates, indicating an antagonistic interaction between moderate light and temperature. The observed increase in carotenoid concentration with rising temperature under both SL and HL conditions likely serves as a protective mechanism against photooxidative damage induced by heat [40]. The results obtained under continuous illumination clearly differ from those under SL conditions (both at 25 µmol m^−2^ s^−1^). Lower levels of total Chl, Chl *a*/Chl *c* ratio, and especially carotenoids, were measured under CL when shifting from 20 °C to 25 °C (Table 1). This suggests that the physiological deregulation induced by continuous illumination [41] alters the response of *P. tricornutum* to higher temperatures.

F_v_/F_m_, a key indicator of the integrity and efficiency of the photosynthetic apparatus, reflects how efficiently light energy is converted into chemical energy by PSII during photosynthesis. The effects of temperature on F_v_/F_m_ have been reported in various marine diatoms [11,42]. F_v_/F_m_ values around 0.6 are expected for healthy *P. tricornutum* cells, while lower values indicate stress caused by biotic or abiotic factors [32]. In our study, F_v_/F_m_ values were not significantly affected by changes in light intensity, although some effects were discernible. Under light/dark cycles, F_v_/F_m_ values slightly increased with temperature (Table 1). The most notable effect was the lowest F_v_/F_m_ values observed under continuous light conditions (Table 1). Additionally, the modest increase in growth observed under CL conditions did not align with the increased irradiance from SL to CL conditions (16 to 24 h of light), indicating a lower efficiency in light use due to the stress induced by the absence of dark periods [41].

The amplitude of the TL signal, related to overall PSII activity from the water-splitting system to the final quinone acceptor [43], was significantly higher at 25 °C compared to 20 °C for the cells grown under both discontinuous illumination conditions (Figure 2B). This result may be attributed to an increased number of photochemically competent PSII complexes in *P. tricornutum* cells at 25 °C. On the other hand, TL experiments also showed that the amount of functional PSII decreased with increasing light intensity at both 20 °C and 25 °C (Figure 2B). This decrease is consistent with the reduction in chlorophyll content at higher light intensities (Table 1), and the consequent loss of reaction centers and FCP complexes [44]. In the case of continuous illumination, the lower F_v_/F_m_ values coincided with similarly lower TL signal values at both temperatures (Figure 2B). This indicates that deregulation induced by continuous illumination led to a similar decrease in the amount of functional PSII complexes in *P. tricornutum* at both temperatures (Figure 2B). A significant effect of temperature on rETR_max_ values was observed (Table 1), with opposite trends in response to the different light regimes tested. Under light/dark cycles in both SL and HL conditions, rETR_max_ values decreased as temperature increased, indicating that the efficiency of electron transport through the photosynthetic apparatus was sensitive to elevated temperatures (Figure 2A and Table 1). A similar pattern has been observed in other diatoms and microalgae [45,46]. The decrease in rETR_max_ beyond the optimum temperature is likely due to the deactivation of carbon fixation processes [47]. Furthermore, under both SL and HL conditions, rETR values decreased similarly at the highest light intensity tested (830 µmol m^−2^ s^−1^) (Table 1), suggesting that the light sensitivity of *P. tricornutum* increases with temperature under light/dark cycles. In contrast, under CL illumination conditions, values for both rETR_max_ and rETR at PAR_max_ increased with temperature (Table 1), indicating that a continuous illumination enhances photosynthetic activity at higher temperature.

On the other hand, PSI efficiency, measured as Y(I) (the fraction of energy absorbed by PSI used in photochemistry), was sensitive to both temperature and light conditions. A reduced ability of PSI to efficiently accept electrons as temperature increased from 20 °C to 25 °C under light/dark cycles was detected. This fact is reflected in the observed decrease in Y(I) and the increase in Y (ND) (the fraction of energy dissipated non-photochemically due to limitations on the donor side of PSI) (Figure 3). This effect may be attributed to a deficiency of PSI donors, leading to an inability to reduce P700 [32,33]. However, the limitation of the donor side of PSI should not be interpreted as a loss of PSI activity. In fact, a photoprotective mechanism of PSI (donor-side regulation) has been proposed, based on the downregulation of the cytochrome *b_6_f* complex through photosynthetic control by luminal acidification. This mechanism decreases the rate of electron transport from PSII to PSI, thereby preventing the over-reduction of P700 [48].

Finally, our data indicate that the photochemical activities of both PSII (measured by TL) and PSI (determined as the maximum P700^+^ signal) were higher at 25 °C (Figure 2B and Table 1). Thus, the observed lower PSI activity was not due to the lack of active PSI centers, but rather to limitations on the PSI donor side. Additionally, P_m_ values decreased with increasing light intensity (at both 20 °C and 25 °C), particularly under continuous light conditions, which can be correlated with the loss of reaction centers and FCP complexes under higher illumination.

### 3.2. Combined Effect of Temperature and Light Regimes on Lipid Content and Fatty Acid Composition

Due to their high lipid and protein content, diatoms can be a valuable source of animal feed, particularly in aquaculture. Additionally, they are considered promising candidates for biofuel production due to their ability to accumulate significant amounts of lipids, including both polar (phosphoglycerides) and non-polar (acylglycerols, sterols, sterol esters, and free fatty acids) lipids [12,49]. When exposed to stress or unfavorable environmental conditions, diatoms often modify their lipid composition, notably by accumulating TAGs, the primary neutral and storage lipids in diatoms. These lipids serve as carbon and energy reserves [20]. Environmental factors, such as light, temperature, nutrient starvation, salinity, and pH changes, can stimulate TAGs accumulation in diatom [49,50]. However, research on the combined effects of temperature and light regime on lipid content and composition in diatoms remains limited [23,24,51,52]. High light intensity generally decreases polar lipid content while promoting an increase in neutral storage lipids, mainly TAGs, which accumulate in LDs [49]. Although LDs are predominantly composed of TAGs, they can also contain SEs and other minor compounds [22].

In this study, the TFA content (related to dry weight) decreased with increasing temperature under the same light regime, while it increased with higher irradiance at each temperature (Figure 4). The observed decrease in PUFA percentage, mainly due to a reduction in EPA, alongside an increase in MUFAs with rising temperature, aligns with previous findings [53]. The increase in TAGs content at 25 °C under HL and, particularly, CL conditions (Figure 4 and Supplementary Figure S2) is consistent with other studies on microalgae, which report enhanced TAG accumulation at elevated temperatures [51]. The increased temperature added additional stress to continuous light exposure, promoting the accumulation of cytoplasmic LDs, even under the moderate light intensity used in our experiments (Figure 1). Continuous illumination at 25 °C not only induced the physiological deregulation of diatom metabolism, but it also inhibited the remobilization of storage lipid due to the absence of dark periods [22]. Interestingly, both temperature and light intensity increase the SE content under light/dark cycles, while a minor effect was observed under CL conditions (Figure 4). Increased sterol levels in response to high light have also been observed in other microalgae, where light-induced stress activates genes involved in sterol metabolism [54].

It is well documented that lipid accumulation, as well as the composition and content of PUFAs and SFAs, vary across different diatom species, growth stages, and environmental conditions [55]. The effect of temperature on the fatty acid composition of marine microalgae is well established, with studies showing a significant increase in the degree of saturation as temperature rises [38,56]. In the present study, *P. tricornutum* followed this trend, with the sum of SFAs and MUFAs increasing, while PUFAs decreased as the temperature rose from 20 °C to 25 °C under all light regimes. The most pronounced changes were observed under CL conditions (Figure 5, upper). Notably, the increase in MUFAs was primarily due to palmitoleic acid (C16:1c), while the decrease in PUFAs was mainly attributed to EPA (C20:5n-3) (Supplementary Table S1). It is widely accepted that an increase in the degree of fatty acid unsaturation serves as an acclimation strategy to low-temperature conditions, promoting the increased fluidity of cell membrane phospholipid layers [15].

Our results demonstrate that 25 °C and CL conditions significantly enhance TAGs accumulation in *P. tricornutum* cells, until they constitute up to 90% of total fatty acids (Supplementary Tables S1 and S2) [57]. These findings are consistent with previous studies that highlight the temperature-dependent modulation of lipid biosynthesis in diatoms [9,10]. Furthermore, the measured TAG contents (in µg mg^−1^ DW) under the different growth conditions tested (Figure 4) were comparable with values reported in other studies [51,54]. The ratios of SFAs, MUFAs, and PUFAs in TAGs were affected by both temperature and light intensity (Figure 5, lower; and Supplementary Table S2). The temperature-dependent trends observed for TFA composition under SL and CL conditions (at the same light intensity) were similarly reflected in TAGs (Figure 5, lower). Palmitoleic (C16:1c) and palmitic (C16:0) acids were the predominant components in TAGs under all light regimes, accounting for up to 88% under CL conditions (Supplementary Table S2). Interestingly, EPA (C20:5n-3) was present only in trace amounts in TAGs (Supplementary Table S2), correlating with the observed decrease in PUFA content under the experimental conditions tested (Figure 5, lower).

## 4. Materials and Methods

### 4.1. Microalgal Strain and Culture Conditions

Cells from the coastal pennate diatom *Phaeodactylum tricornutum* CCAP 1055/1 were grown in artificial seawater (ASW) medium [58] in 250 mL Erlenmeyer flasks (50 mL culture volume) in rotatory shakers (100 rpm) at 20 °C. The cultures were regularly transferred to fresh media and illuminated by LED white light (4500 K) lamps providing an intensity of 25 µmol m^−2^ s^−1^ (T8-150MWBL led lamps, Wellmax) following a light/dark cycle of 16/8 h as standard conditions. To study the effects of temperature and light intensity, cells were grown with controlled temperature (20 °C or 25 °C) for 15 days (at 25 µmol m^−2^ s^−1^ or 60 µmol m^−2^ s^−1^). The 25 µmol m^−2^ s^−1^ light intensity was set as moderate radiation [10] under both light/dark cycles (standard light; SL) and continuous light (CL) conditions. The 60 µmol m^−2^ s^−1^ light intensity (higher light; HL) was set as close to light saturation [10,27]. Cultures were placed at the appropriate distance to achieve the required light intensity, with irradiance being monitored using a LI-250A light meter (LI-COR). Experiments were carried out at two different temperatures—20 °C and 25 °C, corresponding to optimal and higher temperature conditions, respectively. Cultures were inoculated with an initial cell concentration of ≈5.5 × 10^5^ cells mL^−1^. Culture cells were observed and photographed using a Leica microscope DM6000B (IBVF Microscopy Service) when required.

### 4.2. Analytical Methods

Cell growth parameters were obtained from at least ten independent experiments. Cells were counted with a Neubauer-improved hemocytometer (Marienfeld-Superior), following the instructions of the manufacturer. Specific growth rates (µ, day^−1^) were calculated during the exponential phase of growth of cultures using the equation [µ = ln (N_t_/N_0_) /∆t], where N_t_ and N_0_ are the final and initial cell concentrations, respectively, and ∆t is the duration of the growth period in days. Pigments extraction was performed with glass beads (0.5 mm) and 90% cold acetone, as described by [59], with minor modifications. Chlorophyll (Chl) *a* and *c* contents in *P. tricornutum* cells were estimated as described by [60], with the total Chl content being calculated as the sum of Chl *a* and Chl *c*. Carotenoids concentration was determined using the equation provided by [61], as follows:

Chl *a* (µg mL^−1^) = 11.47 × (A_664_ − A_750_) − 0.40 × (A_630_ − A_759_)

Chl *c* (µg mL^−1^) = 24.34 × (A_630_ − A_750_) − 0.40 × (A_664_ − A_759_)

Carotenoids (µg mL^−1^) = 7.6 × (A_480_ − A_750_) − 1.49 × (A_510_ − A_750_)

### 4.3. Nile Red Staining and Fluorescent Microscopy

Nile Red (NR) staining was used to visualize neutral lipids, following the method described by [29] with minor modifications. Briefly, aliquots of *P. tricornutum* cell cultures grown under different irradiance and temperature conditions were collected by centrifugation at 5000 g for 5 min and resuspended in ultrapure water. The cells were then stained with NR (0.5 mg mL^−1^ stock solution in DMSO) to a final concentration of 5 μg mL^−1^ and incubated in the dark for 20 min at 37 °C. NR fluorescence was observed with a fluorescence microscope (Leica DM6000B) with a 100× oil-immersion objective with DIC optics or wide-field fluorescence equipped with a Leica L5 filter cube (excitation bandpass, 480/40 nm; dichroic 505 nm; emission bandpass, 527/30 nm). Images were captured with a digital camera (ORCA-ER, Hamamatsu, Japan).

### 4.4. Lipid Analysis

Total lipids were extracted from *P. tricornutum* cells grown under different irradiance and temperature conditions, following the method described by [62] with some modifications. Briefly, 0.1–0.3 g of dry weight (DW) of *P. tricornutum* cells were homogenized in a chloroform:methanol:water (1:2:2) mixture and subjected to ultrasonic treatment for 20 min in ice water. The samples were then centrifuged for 5 min at 5000× *g* to separate the two phases. The lower chloroform layer containing the lipids was collected. To the upper layer, which contained the methanol:water mixture, 5 mL of distilled water was added to extract the remaining lipids. The solution was vortexed for 120 s and subjected to a second chloroform:methanol:water extraction. Finally, both chloroform fractions containing the lipids were combined, the solvent was completely removed under a stream of N_2_, and total lipids were dissolved in chloroform for further analysis.

Neutral lipids analysis was performed by separating the total lipids using thin-layer chromatography [63]. Individual lipids were visualized under iodine vapor, and identification was performed by comparison with reference standards. Fatty acid methyl esters of the total lipid fraction and individual lipid classes were produced by acid-catalyzed transmethylation [64] and analyzed by gas chromatography using a GC-MS-QP2010 Plus (Shimadzu, Kyoto, Japan) [65]. Heptadecanoic acid was used as an internal standard to calculate lipid and fatty acid contents in the samples. The results are presented as means (μg per mg of DW) ± SD from three independent biological replicates.

### 4.5. Photosynthetic Measurements

To study the global photosynthetic activity, cells from 2.5 mL of the different cultures were collected by centrifugation and resuspended in fresh culture medium. Oxygen intake and evolution rates were determined from three independent experiments using a Clark-type oxygen electrode (Hansatech). Measurements were carried out at 20 °C, both in the dark and under illumination (215 µmol m^−2^ s^−1^), to establish the net photosynthetic activity per cell.

The Chl *a* fluorescence of PSII and the redox state of P700 (the primary donor of photosystem I) from intact cells were determined at room temperature using a pulse-amplitude modulation fluorometer (DUAL-PAM-100, Walz). Photosynthetic parameters were obtained from at least five independent experiments, carried out essentially as previously described [32,33]. Fluorescence measurements were performed using cell suspensions at a concentration of ≈4.5 × 10^6^ cells mL^−1^, which were dark-adapted for 30 min prior to measurements. The maximum quantum yield of PSII (F_v_/F_m_) and the relative linear electron transport rates (rETR) for each actinic light intensity were determined as described in [32,33]. The redox state of P700 was monitored by measuring changes in absorbance at 830 nm versus 875 nm in cell suspensions at a concentration of ≈4.5 × 10^7^ cells mL^−1^. The maximal P700^+^ signal observed upon full oxidation of P700 (P_m_) was determined by the pre-illumination of cell suspensions with FR light (730 nm) for 10 s, followed by a saturating pulse of red light (635 nm) at 10,000 μmol m^−2^ s^−1^ intensity and 0.2 s duration. Quantum yields of photosystem I (PSI) photochemistry, Y(I), donor side limitations, Y(ND), and acceptor side limitations, Y(NA), were determined according to [32,33].

Thermoluminescence (TL) glow curves of *P. tricornutum* cell suspensions were obtained using a home-built apparatus designed by Dr. Jean-Marc Ducruet for luminescence detection from 1 °C to 80 °C. A detailed description of the system can be obtained elsewhere [32,33]. Data acquisition, signal analysis and graphical simulation were performed as previously described [66,67]. TL measurements were carried out as reported in [32,33]. Typically, *P. tricornutum* cell suspensions were dark-incubated for 2 min at 20 °C, then cooled to 1 °C for 1 min. After this period, cells were illuminated with two saturating single-turnover flashes (separated by 1 s). Luminescence emission was then recorded while warming the samples from 1 °C to 65 °C at a heating rate of 0.5 °C per second. Experiments were performed using suspensions with a cell concentration of ≈4.5 × 10^7^ cells mL^−1^. TL parameters were obtained from five independent measurements.

### 4.6. Statistical Significance Level

The significance of the results from two data sets was analyzed using a two-sample t-test calculator (Welch’s *t*-test) tool (https://www.statskingdom.com; accessed on 2 October 2024). The data inputs included means, standard deviations, and N (the number of measurements per group). Differences between data sets were considered statistically significant if the *p*-value was < 0.05.

## 5. Conclusions

Our results underscore the critical role of the interaction between temperature and light intensity in shaping the physiology of *P. tricornutum*. Photosynthetic measurements revealed that *P. tricornutum* exhibits significantly higher light sensitivity as growth temperature increases under light/dark cycles, this trend being reversed under continuous light conditions. Furthermore, while increasing light intensity exacerbated the effects of temperature on PSI activity under light/dark regimes, continuous light exposure partially mitigated the impact of higher temperatures.

Additionally, this study also demonstrates that increasing the temperature from 20 °C to 25 °C, combined with increasing the light intensity under light/dark or continuous light conditions, promotes lipid accumulation, primarily in the form of TAGs, and alters the fatty acid composition in *P. tricornutum* cells. Furthermore, this temperature shift led to an increase in both the number and size of cytoplasmic LDs under HL conditions, with an even more pronounced effect under CL conditions. The most significant finding of this study is that the combination of elevated temperature (25 °C) and continuous illumination caused a drastic increase in TAGs and a shift in their composition, meaning they could be considered for use in biodiesel production.

## Figures and Tables

**Table 1:** General physiological and biochemical parameters of *P. tricornutum* cells and cultures grown under the different temperature and light intensity conditions investigated here.

	SL (25 µmol m^−2^ s^−1^)		HL (60 µmol m^−2^ s^−1^)		CL (25 µmol m^−2^ s^−1^)	
Parameter ^a^	20 °C	25 °C	20 °C	25 °C	20 °C	25 °C
Specific growth rate, µ (day^−1^)	0.294 ± 0.021 ^c^ (100%) ^b^	0.218 ± 0.001 ^c^ (74%)	0.311 ± 0.021 ^c^ (100%)	0.290 ± 0.001 ^c,d^ (93%)	0.317 ± 0.018 ^c,d^ (100%)	0.266 ± 0.001 ^c,d^ (84%)
Cells per mL (×10^7^)	1.680 ± 0.180 ^c^ (100%)	1.250 ± 0.003 ^c^ (74%)	1.880 ± 0.201 ^c,d^ (100%)	1.550 ± 0.004 ^c,d^ (82%)	1.120 ± 0.120 ^c,d^ (100%)	1.210 ± 0.003 ^c,d^ (108%)
Dry weight (µg mL^−1^)	370 ± 61 (100%)	350 ± 54 (94%)	405 ± 68 (100%)	430 ± 10 ^d^ (106%)	310 ± 53 (100%)	340 ± 47 (109%)
Net photosynthetic rate (µmol O_2_ h^−1^ per 10^6^ cells)	35.54 ± 0.18 ^c^ (100%)	15.45 ± 0.09 ^c^ (43%)	22.31 ± 1.45 ^c,d^ (100%)	17.69 ± 0.97 ^c,d^ (79%)	36.67 ± 1.67 ^c^ (100%)	16.93 ± 0.45 ^c,d^ (46%)
Total Chl per cell (pg cell^−1^)	0.85 ± 0.06 (100%)	0.86 ± 0.07 (102%)	0.61 ± 0.02 ^c,d^ (100%)	0.82 ± 0.03 ^c^ (135%)	1.03 ± 0.05 ^c,d^ (100%)	0.82 ± 0.03 ^c^ (79%)
Chl *a*/Chl *c*	4.2 (100%)	6.2 (150%)	6.0 (100%)	8.3 (137%)	3.3 (100%)	2.6 (78%)
Total carotenoids per cell (pg cell^−1^)	0.260 ± 0.004 ^c^ (100%)	0.287 ± 0.001 ^c^ (110%)	0.245 ± 0.001 ^c,d^ (100%)	0.512 ± 0.001 ^c,d^ (209%)	0.361 ± 0.009 ^c,d^ (100%)	0.140 ± 0.004 ^c,d^ (39%)
F_v_/F_m_	0.61 ± 0.02 ^c^	0.66 ± 0.01 ^c^	0.59 ± 0.01 ^c,d^	0.64 ± 0.02 ^c,d^	0.59 ± 0.07	0.58 ± 0.04 ^d^
rETR_max_	6.75 ± 0.18 ^c^	5.17 ± 0.13 ^c^	11.82 ± 0.22 ^c,d^	7.67 ± 0.55 ^c,d^	7.66 ± 0.19 ^c,d^	10.54 ± 0.39 ^c,d^
rETR (at PAR_max_)	3.44 ± 0.51 ^c^	1.88 ± 1.13 ^c^	11.35 ± 0.63 ^c,d^	6.79 ± 0.69 ^c,d^	6.89 ± 0.41 ^c,d^	10.55 ± 0.18 ^c,d^
P_m_	0.24 ± 0.02 ^c^	0.43 ± 0.01 ^c^	0.18 ± 0.02 ^c,d^	0.25 ± 0.01 ^c,d^	0.12 ± 0.02 ^c,d^	0.24 ± 0.01 ^c,d^

^a^ See Section 4 for more details. Specific growth rates (µ) were calculated in the exponential phase of culture growth, while the rest of the parameters were determined after 15 days of culture growth. ^b^ Below, in parentheses, the values are percentages of that of cells grown at 20 °C under each condition. F_v_/F_m_, maximum quantum yield of PSII. rETR_max_, relative maximum electron transport rate. rETR, relative electron transport rate at the maximum photosynthetically active radiation intensity (PAR_max_) of 830 µmol m^−2^ s^−1^. P_m_ maximal P700^+^ signal upon full oxidation. SL, light/dark cycle illumination at 25 µmol m^−2^ s^−1^; HL, light/dark cycle illumination at 60 µmol m^−2^ s^−1^; CL, continuous illumination at 25 µmol m^−2^ s^−1^. ^c,d^ Letters indicate statistically significant differences between treatments (*p* < 0.05; see Section 4). ^c^ Significant differences between the values at 20 °C and 25 °C under the same illumination condition. ^d^ Significant differences between values under SL conditions and those of HL or CL conditions at the same temperature.

## Data Availability

The data that support the findings of this study are available from the corresponding author upon reasonable request.

## Supplementary Materials

The following supporting information can be downloaded at: https://www.mdpi.com/article/10.3390/plants14030329/s1, Figure S1. Thermoluminescence band emissions of *P. tricornutum* cultures grown under standard illumination conditions at different temperatures. Figure S2. Total fatty acids (TFA), triacylglycerides (TAG), and sterol esters (SE) content in *P. tricornutum* cells. Figure S3. Representative chromatograms obtained from gas chromatography analysis of total fatty acids in *P. tricornutum*. Table S1. Total fatty acid content, composition, and saturation ratio of *P. tricornutum* cells grown under different temperature and light intensity conditions. Table S2. Triacylglycerides content, composition, and saturation ratio of *P. tricornutum* cells grown under different temperature and light intensity conditions.
