# Supplementary material for: Combined Effect of Temperature and Different Light Regimes on the Photosynthetic Activity and Lipid Accumulation in the Diatom Phaeodactylum tricornutum
Source: Plants (Basel). 2025 Jan 22;14(3):329. doi: 10.3390/plants14030329 (PMC11820123; doi:10.3390/plants14030329)
Supplement: Supplementary file 1 [file plants-14-00329-s001.zip › Table_S1.pdf]

Table S1. Total fatty acid content, composition, and saturation ratio in *P. tricornutum* cells grown under different temperature and light intensity conditions.

	SL (25 µmol m^{-2} s^{-1})		HL (60 µmol m^{-2} s^{-1})		CL (25 µmol m^{-2} s^{-1})	
**Temperature**	**20ºC**	**25ºC**	**20ºC**	**25ºC**	**20ºC**	**25ºC**
TFA[a)] (µg mg^{-1})	49.73 ± 4.55	48.09 ± 3.56	59.46 ± 1.78	49.44 ± 5.70	71.83 ± 11.70	55.90 ± 3.70
Fatty acid[b)]:						
C14:0	1.91 ± 0.73 (3.85%)[c)]	1.35 ± 0.30 (2.80%)	0.80 ± 0.31 (1.34%)	0.57 ± 0.20 (1.15%)	1.59 ± 0.76 (2.22%)	1.05 ± 0.20 (1.88%)
C16:0	5.13 ± 0.97 (10.33%)	4.09 ± 0.76 (8.51%)	5.19 ± 0.27 (8.73%)	7.39 ± 1.68 (14.95%)	12.06 ± 3.96 (16.79%)	12.14 ± 1.81 (21.71%)
C16:1c	10.87 ± 2.34 (21.86%)	15.21 ± 2.10 (31.64%)	11.44 ± 0.25 (19.23%)	14.11 ± 4.32 (28.53%)	28.16 ± 5.51 (39.21%)	25.55 ± 3.14 (45.71%)
C16:1t	0.20 ± 0.05 (0.40%)	0.26 ± 0.17 (0.55%)	0.25 ± 0.06 (0.42%)	0.11 ± 0.03 (0.23%)	0.26 ± 0.10 (0.36%)	n.d.
C16:2c	0.39 ± 0.09 (0.79%)	1.63 ± 0.24 (3.38%)	0.44 ± 0.03 (0.74%)	0.49 ± 0.15 (1.00%)	0.97 ± 0.32 (1.35%)	0.77 ± 0.08 (1.37%)
C16:2t	0.75 ± 0.16 (1.50%)	0.71 ± 0.11 (1.48%)	2.25 ± 0.05 (3.78%)	1.13 ± 0.35 (2.29%)	1.85 ± 0.52 (2.57%)	0.91 ± 0.07 (1.63%)
C16:3	3.75 ± 0.90 (7.55%)	4.96 ± 0.76 (10.32%)	4.76 ± 0.47 (8.00%)	2.78 ± 0.87 (5.63%)	4.73 ± 1.02 (6.59%)	2.91 ± 0.22 (5.21%)
C18:0	0.54 ± 0.04 (1.08%)	0.75 ± 0.26 (1.55%)	1.23 ± 0.15 (2.07%)	1.11 ± 0.04 (2.24%)	0.71 ± 0.12 (0.99%)	0.65 ± 0.14 (1.16%)
C18:1	1.37 ± 0.61 (2.76%)	0.92 ± 0.13 (1.91%)	0.54 ± 0.12 (0.91%)	1.29± 0.31 (2.61%)	1.03 ± 0.26 (1.43%)	1.41 ± 0.12 (2.52%)
C18:2	1.90 ± 0.86 (3.82%)	0.40 ± 0.06 (0.83%)	1.64 ± 0.25 (2.76%)	1.06 ± 0.27 (2.15%)	1.69 ± 0.32 (2.36%)	0.65 ± 0.05 (1.17%)
C18:3	1.35 ± 0.57 (2.71%)	0.19 ± 0.03 (0.39%)	1.19 ± 0.26 (2.00%)	0.29 ± 0.08 (0.58%)	0.65 ± 0.15 (0.91%)	n.d.
C20:3c	n.d.	0.18 ± 0.03 (0.37%)	0.61 ± 0.46 (1.02%)	0.37 ± 0.05 (0.74%)	0.24 ± 0.24 (0.33%)	0.13 ± 0.11 (0.23%)
C20:3t	0.40 ± 0.07 (0.81%)	0.49 ± 0.09 (1.02%)	0.65 ± 0.59 (1.09%)	0.50 ± 0.10 (1.01%)	0.23 ± 0.39 (0.32%)	0.08 ± 0.14 (0.15%)
C20:4	n.d.	0.27 ± 0.03 (0.55%)	0.22 ± 0.29 (0.37%)	0.37 ± 0.05 (0.75%)	0.32 ± 0.31 (0.44%)	0.41 ± 0.04 (0.73%)
C20:5	19.70 ± 3.50 (39.61%)	15.89 ± 2.63 (33.05%)	26.68 ± 1.34 (44.87%)	16.59 ± 3.14 (33.56%)	16.21 ± 2.99 (22.56%)	8.51 ± 0.63 (15.23%)
C22:6	1.06 ± 0.21 (2.14%)	0.54 ± 0.08 (1.11%)	1.41 ± 0.14 (2.38%)	1.07± 0.23 (2.17%)	1.13 ± 0.31 (1.57%)	0.64 ± 0.06 (1.15%)
C24	0.40 ± 0.07 (0.81%)	0.26 ± 0.11 (0.54%)	0.16 ± 0.28 (0.28%)	0.21 ± 0.18 (0.43%)	n.d.	0.09 ± 0. 15 (0.16%)
UFA/SFA[d)]	5.23	6.46	7.06	4.33	4.00	3.01

[a)]Total fatty acid content (TFA; µg per mg of dry weight). [b)]Composition of total fatty acid measured as µg per mg of dry weight. [c)]Below, in parentheses, values referred to the percentage of total fatty acids. [d)]Ratio between unsaturated (UFA) and saturated (SFA) fatty acids. [e)]n.d., no detected.
