# Supplementary material for: Combined Effect of Temperature and Different Light Regimes on the Photosynthetic Activity and Lipid Accumulation in the Diatom Phaeodactylum tricornutum
Source: Plants (Basel). 2025 Jan 22;14(3):329. doi: 10.3390/plants14030329 (PMC11820123; doi:10.3390/plants14030329)
Supplement: Supplementary file 1 [file plants-14-00329-s001.zip › Table_S2.pdf]

Table S2. Triacylglyceride content, composition, and saturation ratio in *P. tricornutum* cells grown under different temperature and light intensity conditions.

	SL (25 µmol m^{-2} s^{-1})		HL (60 µmol m^{-2} s^{-1})		CL (25 µmol m^{-2} s^{-1})	
**Temperature**	**20ºC**	**25ºC**	**20ºC**	**25ºC**	**20ºC**	**25ºC**
TAG[a)] (µg mg^{-1})	21.87 ± 1.58	22.09 ± 2.67	5.86 ± 0.35	23.33 ± 4.35	28.44 ± 2.50	50.42 ± 4.07
Fatty acid[b)]:						
C14:0	1.27 ± 0.09 (5.79%)[c)]	0.93 ± 0.15 (4.22%)	0.41 ± 0.04 (7.01%)	0.89 ± 0.29 (3.80%)	0.46 ± 0.40 (1.62%)	1.02 ± 0.47 (2.03%)
C16:0	5.30 ± 0.99 (24.25%)	4.58 ± 0.06 (20.75%)	2.69 ± 0.22 (45.91%)	8.55 ± 2.31 (36.66%)	7.87 ± 0.90 (27.68%)	15.64 ± 20.7 (31.03%)
C16:1c	7.61 ± 1.17 (34.82%)	11.50 ± 1.96 (52.08%)	1.38 ± 0.24 (23.63%)	11.30 ± 3.73 (48.41%)	16.13 ± 2.36 (56.74%)	28.46 ± 3.47 (56.46%)
C16:1t	n.d.	n.d.	n.d.	n.d.	n.d.	0.07± 0.14 (0.13%)
C16:2c	0.08 ± 0.03 (0.37%)	0.52 ± 0.16 (2.34%)	n.d.	0.30 ± 0.10 (1.28%)	0.39 ± 0.06 (1.38%)	0.72 ± 0.09 (1.43%)
C16:2t	0.09 ± 0.08 (0.41%)	0.12 ± 0.04 (0.54%)	n.d.	0.24 ± 0.12 (1.03%)	0.48 ± 0.06 (1.68%)	0.47 ± 0.05 (0.94%)
C16:3	0.03 ± 0.03 (0.16%)	0.13 ± 0.05 (0.61%)	n.d.	0.08 ± 0.07 (0.34%)	0.23 ± 0.01 (0.79%)	0.53 ± 0.00 (1.05%)
C18:0	0.80 ± 0.11 (3.65%)	0.71 ± 0.12 (3.21%)	0.97 ± 0.07 (16.54%)	0.83 ± 0.05 (3.58%)	0.42 ± 0.09 (1.47%)	0.59 ± 0.00 (1.17%)
C18:1	5.18 ± 0.33 (23.71%)	2.83 ± 0.82 (12.82%)	0.33 ± 0.06 (5.65%)	0.60 ± 0.07 (2.59%)	0.37 ± 0.01 (1.30%)	0.84 ± 0.01 (1.66%)
C18:2	0.63 ± 0.05 (2.88%)	0.28 ± 0.12 (1.25%)	0.04 ± 0.02 (0.67%)	0.16 ± 0.04 (0.70%)	0.33 ± 0.01 (1.15%)	0.29 ± 0.04 (0.58%)
C18:3	0.11 ± 0.09 (0.48%)	n.d.	n.d.	n.d.	n.d.	n.d.
C20:5	0.69 ± 0.08 (3.17%)	0.46 ± 0.17 (2.08%)	0.03 ± 0.07 (0.59%)	0.38 ± 0.12 (1.62%)	0.13 ± 0.01 (0.44%)	n.d.
C22:1	0.07 ± 0.06 (0.32%)	0.02 ± 0.04 (0.11%)	n.d.	n.d.	1.64 ± 0.03 (5.76%)	1.78 ± 0.11 (3.52%)
UFA/SFA[d)]	1.97	2.55	0.44	1.27	2.25	1.92

Parameters were determined after 15 days of culture growth. [a)]Triacylglyceride content (TAG; µg per mg of dry weight). [b)]Composition of triacylglyceride content measured as µg per mg of dry weight. [c)]Below, in parentheses, values referenced to the percentage of total triacylglyceride content. [d)]Ratio between unsaturated (UFA) and saturated (SFA) fatty acids. [e)]n.d., no detected.
