# Supplementary figures and images for: Combined Effect of Temperature and Different Light Regimes on the Photosynthetic Activity and Lipid Accumulation in the Diatom Phaeodactylum tricornutum
Source: Plants (Basel). 2025 Jan 22;14(3):329. doi: 10.3390/plants14030329 (PMC11820123; doi:10.3390/plants14030329)

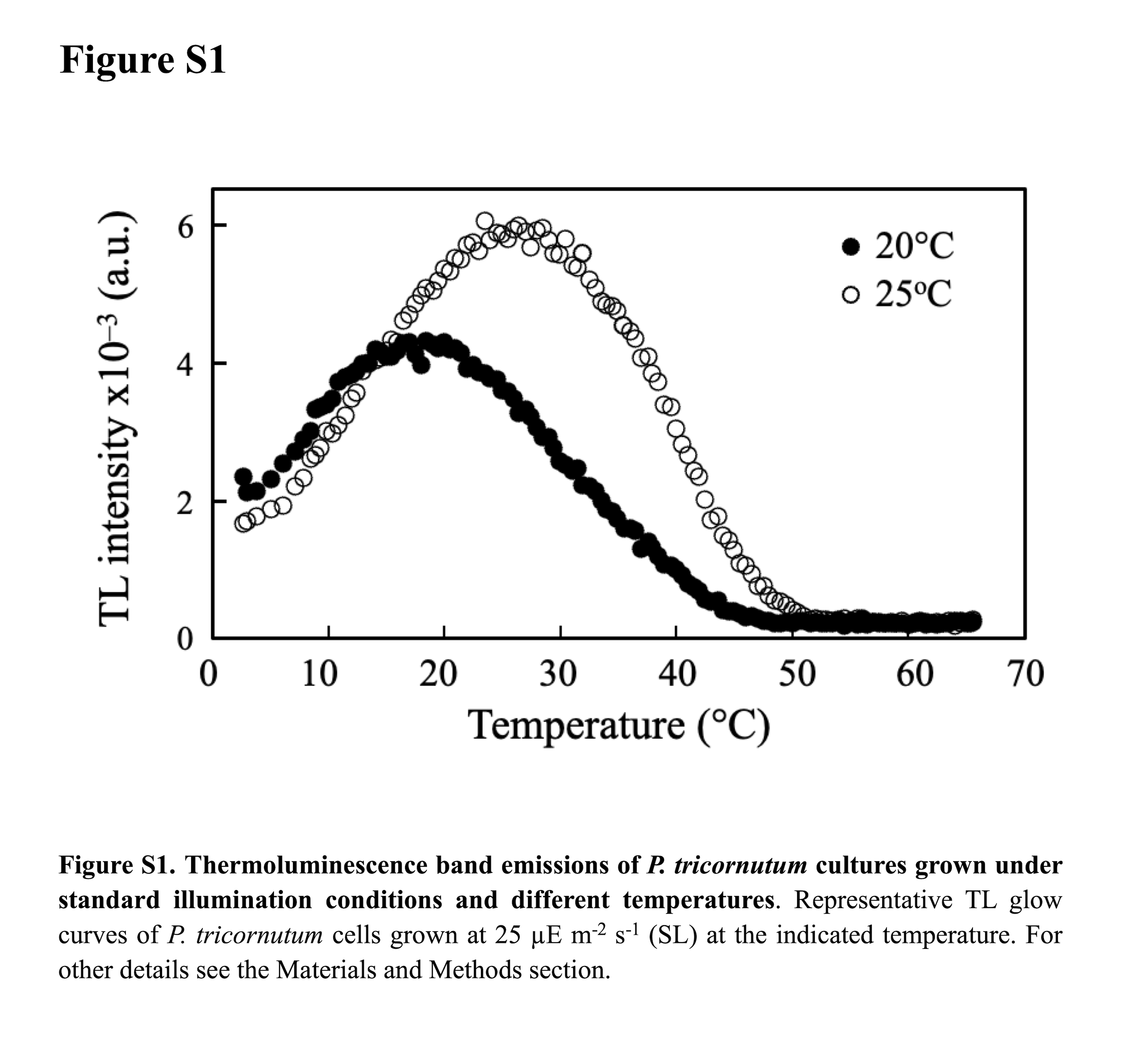

Supplement: Supplementary file 1 [file plants-14-00329-s001.zip › Figure_S1.tiff]

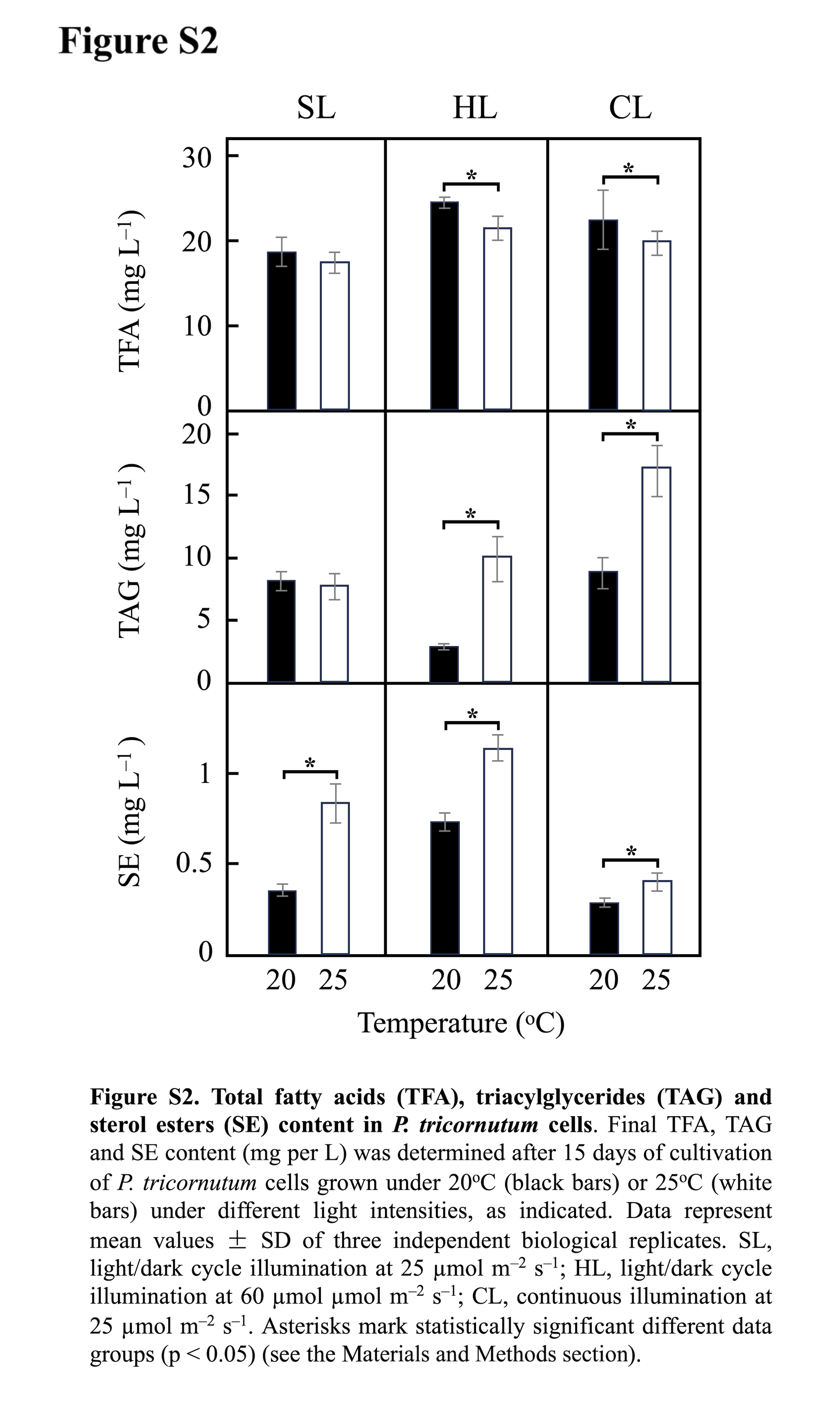

Supplement: Supplementary file 1 [file plants-14-00329-s001.zip › Figure_S2.tiff]

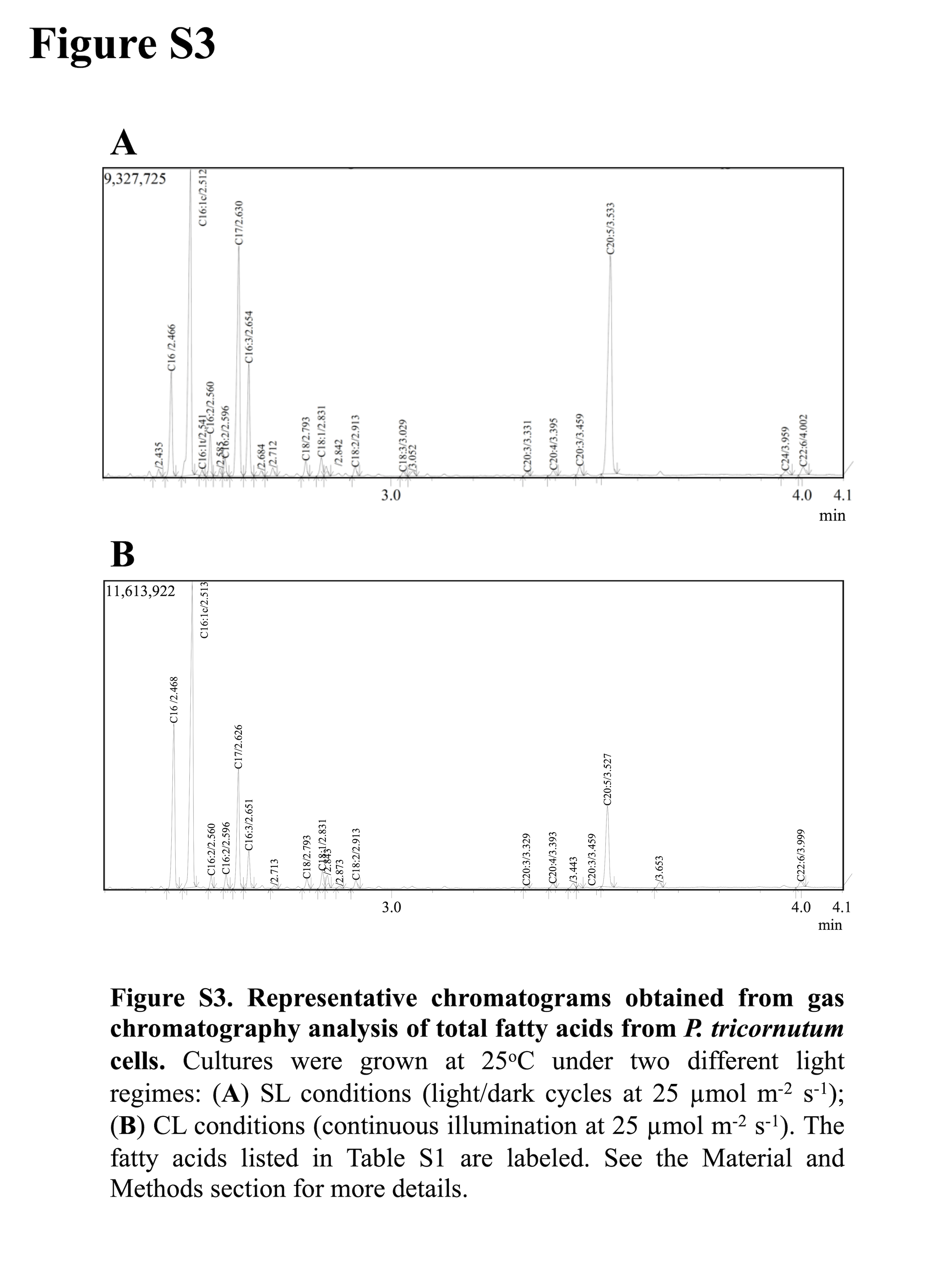

Supplement: Supplementary file 1 [file plants-14-00329-s001.zip › Figure_S3.tiff]
